# The CCR4-NOT complex is a tumor suppressor in *Drosophila melanogaster* eye cancer models

**DOI:** 10.1186/s13045-018-0650-0

**Published:** 2018-08-25

**Authors:** Carmen Vicente, Rocco Stirparo, Sofie Demeyer, Charles E. de Bock, Olga Gielen, Mardelle Atkins, Jiekun Yan, Georg Halder, Bassem A. Hassan, Jan Cools

**Affiliations:** 1 Center for Cancer Biology, VIB, Leuven, Belgium; 2 Center for Human Genetics, KU Leuven, Herestraat 49, box 912, B-3000 Leuven, Belgium; 3 Department of Oncology, KU Leuven, Leuven, Belgium; 4 Center for Brain & Disease Research, VIB, Leuven, Belgium; 5 Institut du Cerveau et de la Moelle Epinière (ICM) - Hôpital Pitié-Salpêtrière, UPMC, Sorbonne Universités, Inserm, CNRS, Paris, France; 6 Centro de Investigación Médica Aplicada, Av. de Pío XII, 55, 31008 Pamplona, Spain

**Keywords:** CCR4-NOT, Leukemia, mRNA stability, Tumor suppressor, *Drosophila melanogaster*

## Abstract

**Background:**

The CNOT3 protein is a subunit of the CCR4-NOT complex, which is involved in mRNA degradation. We recently identified CNOT3 loss-of-function mutations in patients with T-cell acute lymphoblastic leukemia (T-ALL).

**Methods:**

Here, we use different *Drosophila melanogaster* eye cancer models to study the potential tumor suppressor function of *Not3*, the CNOT3 orthologue, and other members of the CCR4-NOT complex.

**Results:**

Our data show that knockdown of *Not3*, the structural components *Not1/Not2*, and the deadenylases *twin/Pop2* all result in increased tumor formation. In addition, overexpression of *Not3* could reduce tumor formation. *Not3* downregulation has a mild but broad effect on gene expression and leads to increased levels of genes involved in DNA replication and ribosome biogenesis. *CycB* upregulation also contributes to the *Not3* tumor phenotype. Similar findings were obtained in human T-ALL cell lines, pointing out the conserved function of Not3.

**Conclusions:**

Together, our data establish a critical role for *Not3* and the entire CCR4-NOT complex as tumor suppressor.

**Electronic supplementary material:**

The online version of this article (10.1186/s13045-018-0650-0) contains supplementary material, which is available to authorized users.

## Background

The CCR4-NOT complex is an essential and conserved multi-subunit complex that regulates gene expression [1]. Although it is implicated in many different cellular functions, it has been mainly studied for its mRNA deadenylation activity, a first step in mRNA degradation [1, 2]. In humans, the CCR4–NOT complex consists of at least nine conserved “canonical” subunits: CNOT1, CNOT2, CNOT3, CNOT6, CNOT6L, CNOT7, CNOT8, CNOT9, and CNOT10. Among these subunits, CNOT6, CNOT6L, CNOT7, and CNOT8 have deadenylase activity and are directly responsible for the removal of the poly-A tail from the target mRNA [3].

Besides, other subunits of the complex are also important for mRNA degradation. For instance, deadenylation is suppressed by *CNOT1* depletion and *CNOT2* downregulation affects the length of mRNA poly-A tails [4, 5]. Recent data indicate that CNOT3 is also involved in the control of mRNA stability. *Cnot3* haplodeficiency in ob/ob mice ameliorated the obese phenotype through the regulation of the CCR4–NOT-mediated deadenylation of specific mRNAs involved in energy metabolism [6]. Furthermore, CNOT3 regulates bone mass through regulation of *Rank* mRNA stability [7]. Recently, it has been shown that CNOT3 contributes to early B cell development by controlling *Igh* rearrangement and *p53* mRNA stability [8].

Escape from post-transcriptional regulation of gene expression is a crucial step in the pathogenesis of cancer. Aberrant polyadenylation site usage, leading to a truncated 3′UTR, has been detected in many human malignancies and might allow malignant cells to escape regulation by both microRNA and RNA binding proteins [9, 10]. The RNA-binding protein TTP has been shown to impair MYC-driven lymphoma development [11]. Deletion of the genes *Zfp36L1* and *Zfp36L2*, which encode RNA-binding proteins, was shown to cause T-cell acute lymphoblastic leukemia (T-ALL) in mice due to impaired *Notch1* mRNA degradation [12].

We recently identified loss-of-function mutations on the *CNOT3* gene in patients with T-ALL [13]. Other studies have confirmed *CNOT3* mutations in T-ALL and have also identified mutations in *CNOT1* and *CNOT2* [14–16]. Furthermore, T-ALL patients with HOXA-rearrangements and terminal 5q deletions show CNOT6 downregulation and high incidence of CNOT3 mutations [17]. These data suggest that the CCR4-NOT complex is involved in cancer, although it remains unclear how it is contributing to tumor development.

Here, we explored how loss of *Not3*, the CNOT3 orthologue, is involved in tumor development using loss-of-function and gain-of-function analyses in *Drosophila melanogaster* eye cancer models. We established that *Not3* behaves as a tumor suppressor gene. Reduction of *Not3* expression resulted in a significant increase in tumor incidence, while its overexpression suppressed tumor formation. Downregulation of other subunits of the CCR4-NOT complex also enhanced tumor formation. Our results indicate that the entire complex and its deadenylase activity are required for tumor suppression, which is linked with *CycB* upregulation and the regulation of genes implicated in DNA replication and ribosome biogenesis.

## Methods

### Fly husbandry

All crosses were raised on standard fly food at 25 °C. All fly lines used are listed in Additional file 1: Table S1. For the generation of eyeless>UAS-RNAi Not3, eyeless>UAS-Dl>UAS-RNAi Not3 and eyeless>UAS-Dl>UAS-RNAi twin animals standard methods were used to recombine UAS-RNAi Not3 (VDRC KK102144, v105990) or UAS-RNAi twin (VDRC KK108897, v104442) with an eyeless-Gal4 or an eyeless-Gal4>UAS-Dl insertion on the second chromosome. To perform experiments for quantifying proliferation, apoptosis, and differentiation, control crosses were established using eyeless>UAS-Dl stock virgins and males carrying UAS-RNAi *white*. This chromosome was maintained in a stock balanced over CyO, GFP. Three independent crosses were established per experimental condition tested, and five transfers from each cross into new fly food flasks were performed.

### Analyses of eye tumor burden

Adult animals of the correct genotypes were imaged using a Zeiss Apotome microscope. To analyze tumor burden, each eye was scored separately on flies with the genotype of interest (positive F1 progeny). The score of the eye tumor burden was performed double-blind (except for those experiments in which we investigated the function of the seven selected target genes, shown in Fig. 6). Eyes were counted as hyperplastic when showing at least one fold. Metastases were observed as masses of amorphous red-pigmented cells outside of the eye field (head, thorax, and abdomen). The percentages shown on the bar graphs represent the average percentage of three independent crosses, and the mean number of eyes analyzed is indicated on each graph (*Y*-axis). In the experiments involving (1) the downregulation of *Not3/twin* on the sensitized, the *Ras*-V12, or the wild-type genetic backgrounds and (2) the overexpression of *Not3* or the *Not3* mutant construct on the eyeful genetic background, the whole eye area (region with presence of differentiated photoreceptors) was measured on representative adult eyes. Image processing and eye measurements were performed with ImageJ. Control eyes measured values were considered as 100%. Values for eight individual discs were plotted. Data was analyzed using GraphPad Prism v6.

### Immunohistochemistry, imaging, and quantification

Dissections and stainings were performed as previously described [18]. Primary antibodies used were rat anti-ELAV (1:100, DSHB), rabbit anti-Phospho-Histone-3 (1:1000, Millipore), and rabbit anti-cleaved-DCP1a (1:150, Cell signaling). All secondary antibodies were used at 1:500. Samples were mounted in Prolong antifade mounting media. Fluorescence imaging was performed using a Leica confocal microscope. Images were processed (Z-projection) using ImageJ/FIJI. Phospho-histone H3 (pH3), cleaved-DCP1a, and BrdU positive cells within the posterior part of eye discs from the appropriate genotypes (GFP negative larvae) were counted. Values for 10 individual discs were plotted. Data was graphed and analyzed using GraphPad Prism v6.

### RNA-sequencing sample preparation and analyses

Dissection tools and surfaces were treated with RNAseq Away, and RNA was isolated using the RNAqueous-Micro Kit (Ambion). Eye-antennal imaginal discs were dissected from 30 L3 wandering larvae and transferred into RNAse-free ependorfs containing lysis buffer on ice. Following dissection, either RNA was extracted immediately or discs on lysis buffer were snap-frozen and kept at − 80 °C for later extraction. All the RNA samples showed high quality on the Bioanalyzer (Agilent Technologies). Next-generation sequencing libraries were constructed from 500 ng of total RNA using the Truseq RNA sample prep kit v2, and RNA-seq libraries were subjected to 1 × 50 bp single-end sequencing on a HiSeq2500 instrument (Illumina). For each condition, three replicates were sequenced. The reads were cleaned with fastq-mcf, and a quality control was performed with FastQC (http://www.bioinformatics.babraham.ac.uk/projects/fastqc). Reads were then mapped to the *Drosophila melanogaster* genome (dm6) with TopHat2 [19]. Subsequently, HTSeq-count [20] was used to count the number of reads per gene. For differential gene expression analysis, the Bioconductor package DESeq2 [21] was used. The lists of differential genes were then further analyzed using FlyMine [22].

### Drosophila Not3 overexpression and S2 cell transfection experiments

All Not3 constructs were cloned into the pUASTattB vector. We used Canton S L3 wandering larval eye discs cDNA as template. Fragments were PCR amplified using the primers shown in Additional file 1: Table S2. All constructs were verified by Sanger sequencing. The transgenic flies were generated by BestGene (Strain #9744, 89E11 acceptor site). Drosophila S2 cells were co-transfected with a pMT-Gal4 plasmid and a flag-tagged version of each of the UAS-Not3 plasmids. Gal4 expression was induced 24 h post-transfection by adding CuSO_4_. Cells were treated with cycloheximide (50 μg/ml, 3 h treatment) 24 h after gene expression induction. Lysates were immunoblotted with anti-Flag antibodies. To test the stability of *Cycb*, *fancl*, or *upd2* mRNAs in Drosophila S2 cells, co-transfections of the pMT-Gal4 plasmid and the RNAi plasmid targeting Not3 (construct ID 4068, dna4068 from the VDRC) or a UAS-YFP plasmid (as a negative control) were performed using an Amaxa nucleofector. Gal4 expression (thus, Not3 RNAi or YFP expression) was induced 24 h post-transfection by adding CuSO4. After 24 h of Not3 RNAi or YFP expression induction, transcription was stopped by addition of 5 μg/ml actinomycin D. Cells were harvested, and RNA was purified after 0, 10, 20, 40, 80, and 160 min and quantified by qPCR. Three independent experiments were performed. Expression of the *CG1239* gene was used as a control of no stabilization, since its expression was not significantly changed on our RNA-sequencing experiments after *Not3* downregulation.

### cDNA synthesis and qPCR analyses

cDNA synthesis from eye discs was performed using the QuantiTec Reverse Transcription kit following manual instructions. cDNA synthesis from S2 cells experiments was performed using the GoScript Reverse Transcription system protocol (Promega). Real-time qPCR reactions were performed using the GoTaq Real-Time kit, and reactions were run in a Lightcycler 480 device (Roche). Primers are listed in Additional file 1: Table S2.

### T-ALL cell lines electroporation and RNA stability assay

Jurkat and CCRF-CEM cells were cultured under standard conditions. Electroporations were performed using the gene pulser Xcell™ electroporation system as previously described [23]. The negative control siRNA (D-001810-01-20), CNOT3 siRNA 326 (J-020319-06), and CNOT3 siRNA 328 (J-020319-08) were purchased from Dharmacon. Cells were treated with 5 μg/ml Actinomycin D (Sigma Aldrich) 24 h after electroporation. RNA was immediately extracted at 0 h, 2 h, and 4 h with Maxwell® simplyRNA Cells Kit. Regarding the RNA-sequencing analysis pipeline, reads were mapped to the human genome (GRCh37/hg19). For the differential gene expression analysis (DESeq2), a linear model was applied, as we needed to compare all conditions with each other (instead of a one-to-one comparison). The data was modeled as TP + KD + TP:KD, in which the term TP (time point) represents the mRNA degradation over time, the term KD (knockdown) is related to the knockdown of CNOT3, and the combined term TP:KD shows the impact of the CNOT3 knockdown on mRNA degradation. The differential genes resulting from this last term, which are the more stable mRNAs (less degraded), were then used for further analysis with DAVID [24].

## Results

### Not3 behaves as a tumor suppressor gene in *Drosophila melanogaster* eye cancer models

**Fig. 1:**
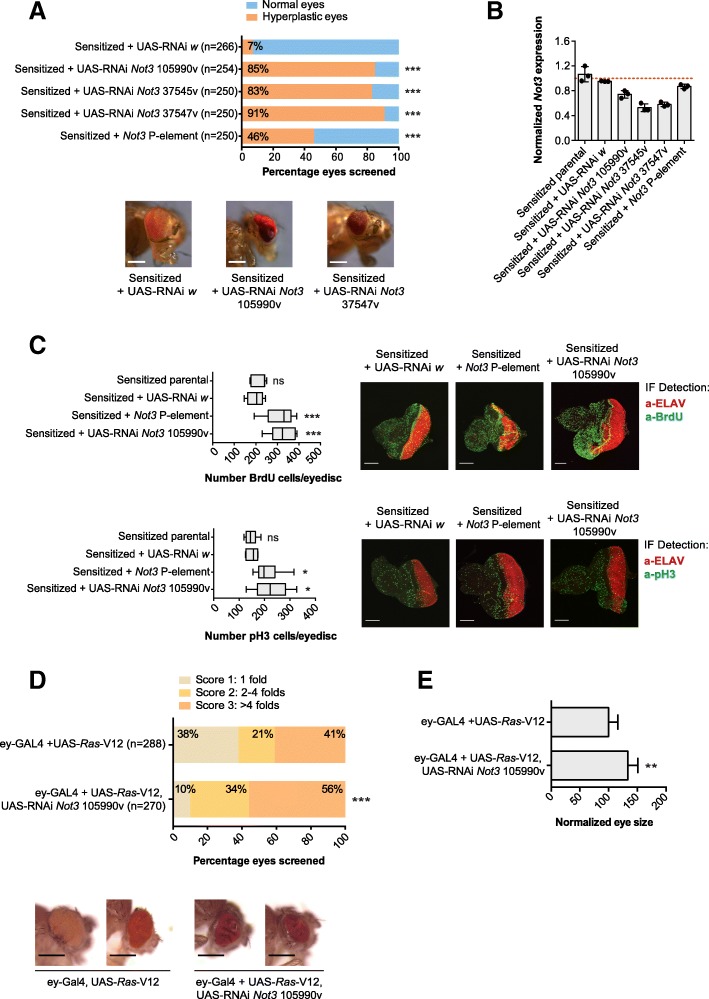
Reduced expression of *Not3* increases tumor formation in *Drosophila melanogaster* eye cancer models. **a** Qualitative and quantitative representation of the tumor burden upon downregulation of *Not3* on sensitized flies. Bars show the percentage of eyes screened: blue, normal eyes; orange, hyperplastic eyes; *** *p* < 0.001. Three independent experimental crosses were established. The mean number of eyes screened is shown on the graph (*Y*-axis). Microscopy images show eyes of adult flies from representative genotypes, scale bars are 200 μM. **b** qPCR analyses showing the *Not3* expression levels from each genotype. Expression values are calculated from three independent crosses. From each cross, 30 eye-antennal imaginal discs were dissected. The red dotted bar represents the expression value = 1. **c** Quantification of BrdU and pH3 positive cells on the posterior portion of eye-antennal imaginal discs. Values for 10 individual discs were plotted. Representative confocal images of eye-antennal imaginal discs of the indicated genotypes. Green staining: BrdU or pH3 positive cells; red staining: ELAV protein. Scale bars are 70 μM; ns, not significant; * *p* < 0.05; *** *p* < 0.001. **d** Qualitative and quantitative representation of the tumor burden upon downregulation of *Not3* on the *Ras*-V12 background. Bars show the average percentage of eyes screened: light orange, eyes classified as score 1 (presence of 1 fold); yellow, eyes classified as score 2 (presence of 2–4 folds); dark orange, eyes classified as score 3 (presence of more than 4 folds); *** *p* < 0.001. Three independent experimental crosses were established. The mean number of eyes screened is shown on the graph (*Y*-axis). Microscopy images show eyes of adult flies from representative genotypes, scale bars are 200 μM. **e** Quantitative representation of the whole eye area (region with presence of differentiated photoreceptors) on representative adult eyes (*n* = 8) with downregulation of *Not3* on the *Ras*-V12 genetic background. Control eyes measured values were considered as 100%; ** *p* < 0.01

To investigate the tumor suppressor role of *Not3*, we downregulated or overexpressed it in various genetic backgrounds. As a first model, we used flies with overexpression of the Notch ligand Delta (*Dl*) in the eye (driven by *ey*-Gal4) which results in hyper-activation of the Notch signaling pathway, thereby, leading to an increase in eye size but no tumor development [18, 25]. We refer to these flies as “sensitized” flies. We downregulated the expression of *Not3* in this genetic background using three different UAS-RNAi Not3 lines, and a line with a P-element transposon insertion, in which the expression of one of the *Not3* alleles has been shown to be disrupted [26]. Reduction of *Not3* expression resulted in a remarkable increase in tumor incidence, from 7% of the eyes with control RNA interference (UAS-RNAi *white*) to up to 90% with the three different *Not3* RNAi lines (Fig. 1a). Strikingly, inactivation of one allele of *Not3* by the P-element insertion (which results in modest downregulation of *Not3*) was sufficient to induce tumor formation in 50% of the eyes (Fig. 1a). In all cases, tumor development was observed without metastases. These data support the hypothesis that loss of *Not3* is sufficient to transform a sensitized lesion into a tumor, possibly by interfering with patterning and cell fate determination.

Next, we performed immunofluorescence stainings on sensitized L3 larval eye discs to investigate changes on the number of proliferative and differentiated cells upon *Not3* downregulation. To do that, we used the phospho-Histone3 (pH3) and BrdU as markers of proliferation and ELAV as a marker of differentiation. Knockdown of *Not3* expression was first confirmed by qPCR (Fig. 1b) and resulted in a significant increase of pH3 and BrdU positive cells (Fig. 1c). Downregulation of *Not3* expression did not cause a significant loss of ELAV expressing cells (differentiated cells), although it did markedly disrupt the pattern of ELAV expression when compared to parental and control eye discs (Fig. 1c).

To further confirm the tumor suppressor activity of *Not3* in a different context, we used a fly model that specifically overexpresses *Ras*-V12 in the eye. Overexpression of only *Ras*-V12 resulted in increased eye size and also in hyperplastic tissue with a penetrance of 62%. Reduction of *Not3* in this oncogenic background resulted in a marked increase in tumor incidence (90%) and more aggressive phenotype as indicated by increased folds observed on the eye (Fig. 1d, e). Thus, reduction of *Not3* expression increases proliferation of retinal precursors in the *Dl*-sensitized and the *Ras*-V12 backgrounds.

### Downregulation of Not3 abrogates normal retinal differentiation

**Fig. 2:**
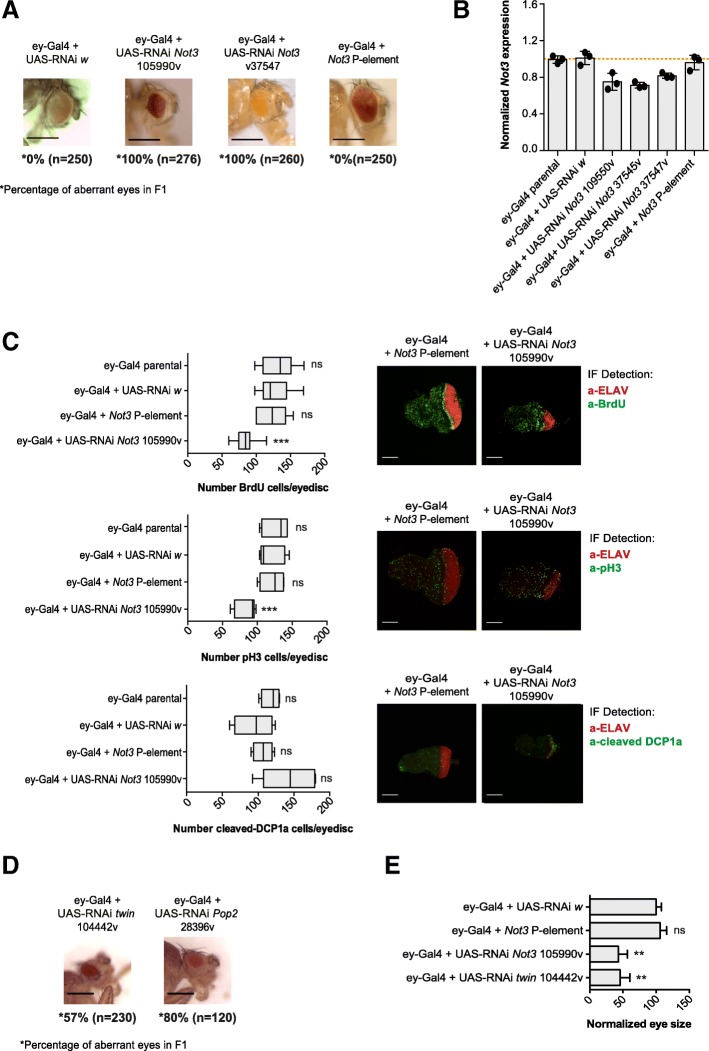
Reduced expression of *Not3* in a wild-type genetic background abolishes normal retinal differentiation. **a** Microscopy images show eyes of adult flies with *Not3* reduced expression levels from representative genotypes, scale bars are 200 μM. The percentage of F1 “small rough” defective eyes and the mean number of eyes screened are indicated on the bottom side of each image. Three independent crosses were performed for each experimental condition. **b** qPCR analyses showing the *Not3* expression levels from each genotype. Expression values are calculated from three independent crosses. From each cross, 30 eye-antennal imaginal discs were dissected. The red dotted bar represents the expression value = 1. **c** Quantification of BrdU, pH3, and cleaved-DCP1a positive cells on the posterior portion of eye-antennal imaginal discs (*n* = 10). Representative confocal images of eye-antennal imaginal discs of the indicated genotypes. Green staining: BrdU, pH3, or cleaved-DCP1a positive cells; red staining: ELAV protein. Scale bars are 70 μM; ns, not significant; *** *p* < 0.001. **d** Microscopy images show eyes of adult flies with downregulation of the *Pop2* and twin CCR4-NOT complex subunits on a wild-type background, scale bars are 200 μM. The percentage of F1 “small rough” defective eyes and the mean number of eyes screened are indicated on the bottom side of each image. **e** Quantitative representation of the whole eye area (region with presence of differentiated photoreceptors) on representative adult eyes (*n* = 8) with downregulation of *Not3* or *twin* on the wild-type genetic background. Control eyes measured values were considered as 100%; ns, not significant; ** *p* < 0.01

We next aimed to dissect the physiological role of *Not3* in eye development. We observed that reduction of *Not3* expression in wild-type eyes (driven by *ey*-Gal4) resulted in a significant decrease of eye size (small rough phenotype) with a penetrance of 100% in adult flies (Fig. 2a). Expression of a UAS-RNAi *white* construct (control) did not change the external morphology of the eye. The *Not3* P-element mutant line showed no obvious defects in retinal differentiation, maybe because the reduction of *Not3* expression levels was not enough to cause defects on a wild-type tissue (Fig. 2b). These data indicate that *Not3* expression is essential for proper retinal development.

We also performed immunofluorescence stainings on larval eye discs to investigate the number of pH3/BrdU, cleaved-DCP1a (marker of apoptosis), and ELAV positive cells. Although not significant, we observed that reduction of *Not3* resulted in less cell proliferation, measured by pH3 and BrdU. Our results also showed that loss of *Not3* led to induction of apoptosis. Differentiation was strongly inhibited, although some photoreceptors could still form in *Not3*-downregulated eye discs (Fig. 2c–e).

Next, we tested if downregulation of other subunits of the CCR4-NOT complex could cause similar effects. Reduction of *twin* (deadenylase, CNOT6/CNOT6L orthologue) or *Pop2* (deadenylase, CNOT7/CNOT8 orthologue) expression led to a phenotype similar to the one observed among *Not3*-defective eye discs (rough small eyes) (Fig. 2d–e, Additional file 2: Figure S1A). Downregulation of *Not1* (scaffold protein of the complex, *CNOT1* orthologue) and *Not2* (*CNOT2* orthologue) was not significant, and as a consequence, the effects of *Not1* or *Not2* downregulation could not be assessed (Additional file 3: Figure S2A).

These results show that reduction of *Not3* or the CCR4-NOT complex subunits beyond a certain threshold disrupts normal retinal differentiation and causes subsequent loss of the differentiated tissue in a wild-type background.

### Overexpression of Not3 suppresses tumor formation

**Fig. 3:**
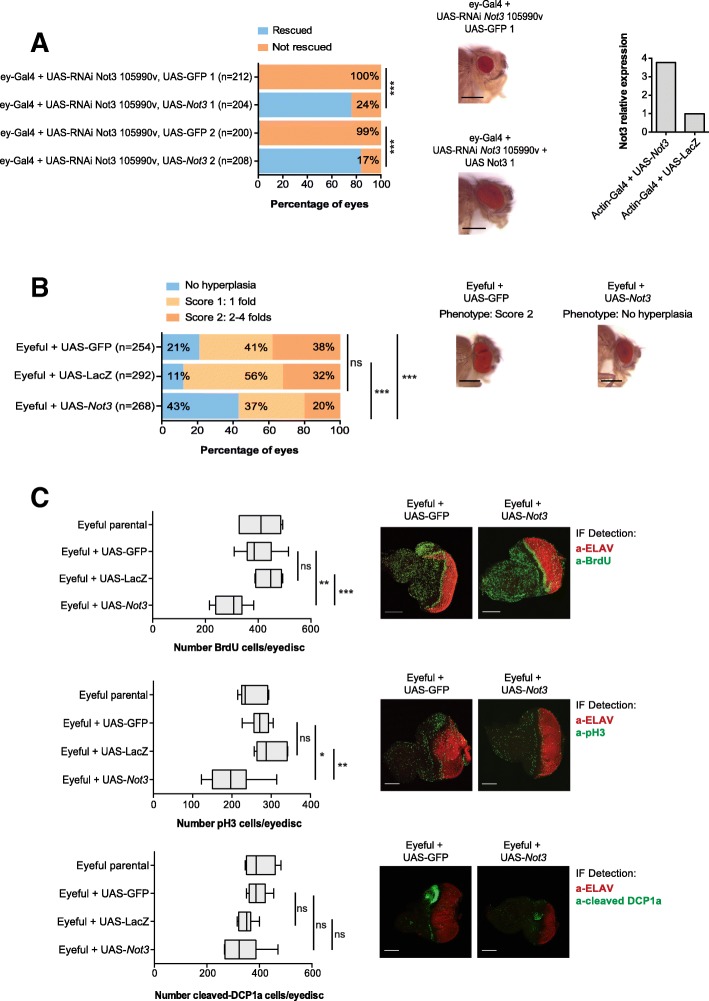
Ectopic expression of *Not3* suppresses tumor formation in eyeful flies. **a** Qualitative and quantitative representation of the defective eyes upon ectopic expression of *Not3* on wild-type flies with reduced *Not3* expression. Bars show the percentage of eyes screened: blue, rescued eyes; orange, not rescued eyes; *** *p* < 0.001. Numbers 1 and 2 on each genotype represented on the graph refer to different recombinant clones. Three independent crosses were analyzed. The mean number of eyes screened is shown on the graph (*Y*-axis). Microscopy images show eyes of adult flies from representative genotypes, scale bars are 200 μM. qPCR analyses showing the *Not3* expression levels from each genotype. Each bar represents the expression value of a pool of 10 individuals. **b** Qualitative and quantitative representation of the hyperplastic eyes upon ectopic expression of *Not3* on eyeful flies. Bars show the percentage of eyes screened: blue, eyes with no hyperplasia; light orange, eyes classified as score 1 (presence of 1 fold); dark orange, eyes classified as score 2 (presence of 2–4 folds); ** *p* < 0.01, *** *p* < 0.001. Three independent crosses were analyzed. The mean number of eyes screened is shown on the graph (*Y*-axis). Microscopy images show eyes of adult flies from representative genotypes. Scale bars are 200 μM. **c** Quantification of BrdU, pH3, and cleaved-DCP1a positive cells on the posterior part of L3 larval eye-antennal imaginal discs. Values for 10 individual discs were plotted. Representative confocal images of eye-antennal imaginal discs of the indicated genotypes. Green staining: BrdU, pH3, or cleaved-DCP1a positive cells; red staining: ELAV protein. Scale bars are 70 μM; ns, not significant; * *p* < 0.05; ** *p* < 0.01; ***p < 0.001

To investigate whether *Not3* overexpression could suppress tumor formation, we first generated transgenic flies carrying a UAS-*Not3* construct. Ectopic expression of *Not3* was able to rescue the small rough eye phenotype previously observed in wild-type *Not3*-downregulated eyes, indicating that both UAS-*Not3* overexpression and the effects observed upon *Not3* downregulation are specific (Fig. 3a).

For subsequent experiments, we used the so-called "eyeful" flies. These flies have overexpression of the Notch ligand *Dl*, together with overexpression of the epigenetic regulators *lola* and *psq*. Eyeful flies display excessively enlarged eyes and eye tumors and macroscopically visible metastases derived from the developing retina [18, 25]. Overexpression of *Not3* in this model significantly suppressed formation of eye tumors: 43% vs. 21% (*p* < 0.01) when compared with UAS-GFP or 43% vs. 14% (*p* < 0.001) when compared with UAS-LacZ. When hyperplastic eyes were detected, the tumorigenic phenotypes observed were also milder than the ones observed in the control crosses (Fig. 3b).

In the eyeful eye discs, disorganization of the epithelium as well as defects in the pattern of differentiated cells were evident (Fig. 3c). Eyeful L3 larval eye discs with *Not3* overexpression had significantly less proliferating cells (less pH3/ BrdU positive cells) than control eye discs (Fig. 3c). Ectopic expression of *Not3* did not significantly affect the number of apoptotic cells (cleaved-DCP1a), although it seemed there were fewer apoptotic cells when compared to the control conditions. In addition, upregulation of *Not3* expression resulted in the re-establishment of the typical epithelial organization, accordingly to ELAV stainings.

Taken together, our data show that *Not3* acts as a classical tumor suppressor in *Drosophila melanogaster* eye cancer models, with its downregulation enhancing tumor formation and its overexpression suppressing tumor formation/progression.

### The tumor suppressor function of Not3 is related to its function within the CCR4-NOT complex

**Fig. 4:**
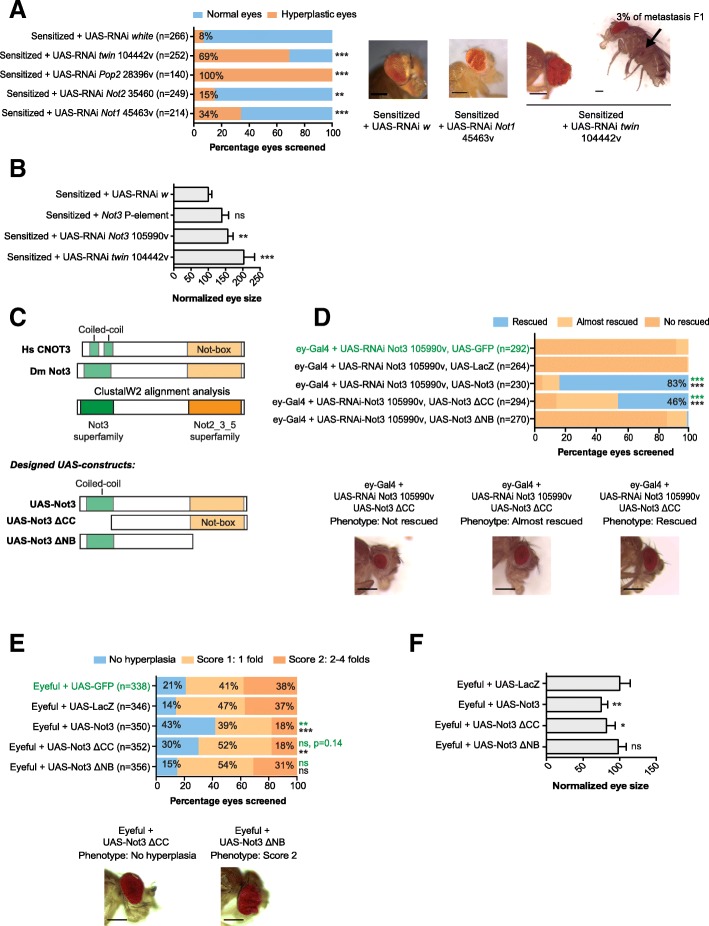
The *Not3* Not-box domain is important for normal protein functionality. **a** Qualitative and quantitative representation of the tumor burden upon downregulation of different CCR4-NOT complex subunits on the sensitized background. Bars show the percentage of eyes screened: blue, normal eyes; orange, hyperplastic eyes; ** *p* < 0.01; *** *p* < 0.001. The mean number of eyes screened is shown on the graph (*Y*-axis) and three independent crosses were established. Microcopy images show the eyes of adult flies from representative genotypes. Scale bars are 200 μM. Black arrow indicates the presence of metastasis. **b** Quantitative representation of the whole eye area (region with presence of differentiated photoreceptors) on representative adult eyes (*n* = 8) with downregulation of *Not3* or *twin* on the sensitized genetic background. Control eyes measured values were considered as 100%; ns, not significant; ** *p* < 0.01; *** *p* < 0.001. **c** Schematic representation of the human CNOT3 and the fly Not3 proteins. ClustalW2 alignment analyses show that the coiled-coil (green) and Not-box (orange) regions are present in both species. Designed UAS constructs are also shown: UAS-Not3 (wild-type Not3), UAS-Not3 ΔCC (deletion of the coiled-coil domain region), UAS-Not3 ΔNB (deletion of the Not-box domain). **d** Qualitative and quantitative representation of the defective eyes upon ectopic expression of Not3, Not3-ΔCC, and Not3-ΔNB on wild-type flies with reduced Not3 expression. The mean number of eyes screened is shown on the graph (*Y*-axis), and three independent crosses were established. Bars show the percentage of eyes screened: blue, normal eyes; light orange, eyes classified as “almost rescued”; dark orange, eyes classified as “not rescued”; *** *p* < 0.001. In green color, comparisons to UAS-GFP control flies; black color, comparison to UAS-LacZ control flies. Microscopy images show the eyes of adult flies from each category. Scale bars are 200 μM. **e** Qualitative and quantitative representation of the defective eyes upon ectopic expression of Not3, Not3-ΔCC, and Not3-ΔNB on eyeful flies. Bars show the percentage of eyes screened: blue, normal eyes; light orange, eyes classified as score 1 (presence of 1 fold); dark orange, eyes classified as score 2 (presence of 2–4 folds); ns, means not significant, ** *p* > 0.01, *** *p* < 0.001. The mean number of eyes screened is shown on the graph (*Y*-axis). In green color, comparisons to UAS-GFP control flies; black color, comparison to UAS-LacZ control flies. Microscopy images show the eyes of adult flies from each category. Scale bars are 200 μM. **f** Quantitative representation of the whole eye area (region with presence of differentiated photoreceptors) on representative adult eyes (*n* = 8) with ectopic expression of *Not3* wild-type *or Not3* mutants on the eyeful genetic background. Control eyes measured values were considered as 100%; ns, not significant; * *p* < 0.05; ** *p* < 0.01

Since *Not3* is a subunit of the CCR4-NOT complex, we asked whether downregulation of other subunits in the sensitized background causes similar effects. Reduction of expression of *Not1*, *Not2*, *twin*, or *Pop2* all caused increased tumor formation (Fig. 4a, b, Additional file 2: Figure S1B, and Additional file 3: Figure S2B). Of interest, reduction of *twin* also caused metastatic tumors (Fig. 4a). These data confirm that not only *Not3*, but also the entire CCR4-NOT complex functions as a tumor suppressor.

*CNOT3*, the human *Not3* orthologue, contains three important domains. The N-terminal region contains two coiled-coil (CC) domains, while the C-terminal region harbors a Not-box domain (NB). ClustalW2 alignment analysis showed that the CC and NB domains are conserved in the Not3 protein from *Drosophila melanogaster* (Fig. 4c). We then characterized which Not3 domains are essential for tumor suppression in the eyeful background. We designed and cloned two versions of the wild-type *Not3* open reading frame in a pUASTattB vector: UAS-*Not3*-ΔCC (lacking the CC domain) and UAS-*Not3*-ΔNB (lacking the NB domain) (Fig. 4c). First, we measured whether the expression levels achieved by ectopic expression of these constructs were similar. Since a good antibody against the Not3 protein from *Drosophila melanogaster* was not available, we co-transfected S2 cells with a pMT-Gal4 plasmid and a flag-tagged version of each of the UAS-Not3 plasmids. Cycloheximide treatment of the transfected cells was also performed in order to investigate the stability of the translated proteins. Our results showed that similar expression levels were achieved with all plasmids and that the translated proteins were equally stable (Additional file 4: Figure S3A).

Next, we engineered transgenic flies expressing the different versions of the wild-type *Not3* open reading frame (without a flag-tag). Since *Not3* is on chromosome 2 on flies, we decided to insert our UAS-*Not3* transgenes on chromosome 3 (site 89E11). Our qPCR analyses showed that ectopic expression of UAS-*Not3* plasmids driven by Actin5C-Gal4 results in similar expression levels among all constructs used in these experiments (Additional file 4: Figure S3B).

Ectopic expression of UAS-*Not3*-ΔCC in *Not3*-defective flies rescued the *Not3* small rough eye phenotype previously observed, while no rescue was observed upon expression of UAS-*Not3*-ΔNB (Fig. 4d). Similar results were observed in the eyeful background. Overexpression of UAS-*Not3*-ΔCC had a mild effect tumor formation with 30% penetrance, while UAS-*Not3*-ΔNB did not suppress the tumor phenotype at all (Fig. 4e, f).

Taken together, our results show that the NB domain is essential to rescue developmental and cancer phenotypes observed upon downregulation of *Not3*. Since the NB domain is essential for the association of *Not3* with the CCR4-NOT complex [27, 28], these data further indicate that the effects observed with downregulation of *Not3* are linked to its function within the CCR4-NOT complex.

### Not3 disruption in the sensitized background causes an aberrant gene expression program

**Fig. 5:**
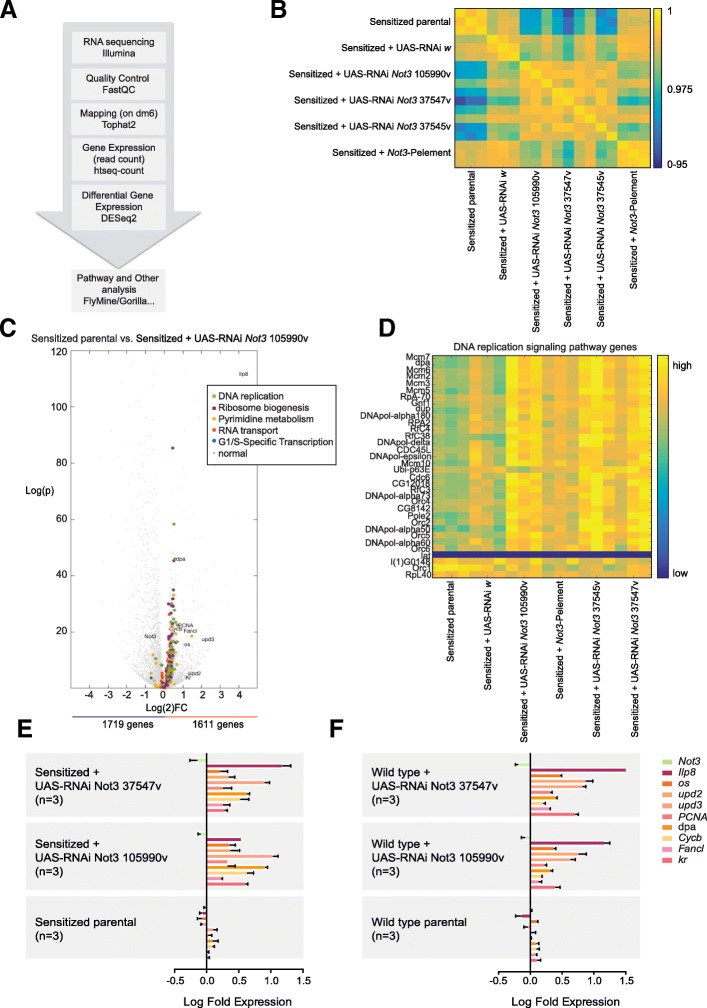
*Not3* reduced expression in the sensitized background drives oncogenic altered gene expression. **a** Workflow for the analysis of the RNA-sequencing experiments performed on fly eye-antennal eye discs. **b** Correlation matrix between the different samples, calculated on the normalized mRNA expression levels. **c** Volcano plot of all differential genes between sensitized + UAS-RNAi *Not3* 105990v and sensitized parental, which represents the results of three biological replicates. The other *Not3* knockdowns show similar results. The genes belonging to one of the most enriched signaling pathways are marked in color. **d** Matrix showing the normalized mRNA expression levels of those genes that belong to the DNA replication signaling pathway. **e** qPCR validation experiments of selected genes on the sensitized + *Not3* defective tumor models. Data were normalized accordingly to the expression levels on the sensitized + UAS-RNAi *white* eye-antennal discs. Results are plotted as mean ± standard deviation. **f** qPCR validation experiments of selected genes on the ey-Gal4 *Not3* defective models. Data were normalized accordingly to the expression levels on the ey-Gal4 + UAS-RNAi *white* eye-antennal discs. Results are plotted as mean ± standard deviation

The major role of the CCR4-NOT complex is to regulate mRNA stability through deadenylation of mRNA. To determine which mRNA transcripts are affected by *Not3* knockdown, we carried out RNA-sequencing analysis (Fig. 5a) on L3 larvae eye discs isolated from the sensitized parental, sensitized control (sensitized + UAS-RNAi *white*), and sensitized *Not3-tumor* eye discs: sensitized + *Not3*-Pelement and sensitized + UAS-RNAi *Not3* knockdowns. Correlations were calculated between the mRNA expression profiles of the different samples. High correlations were observed between the different *Not3* knockdown samples. The sensitized + *Not3* P-element samples were more closely related to the control samples, which is in agreement with mild reduction of *Not3* in this model and mild phenotypic effects observed (Fig. 5b).

A differential gene expression analysis was performed between the different *Not3* knockdown models and the sensitized parental, confirming the significant downregulation of *Not3* mRNA levels with an average log2 fold change of − 0.2270. Moreover, these analyses revealed that approximately 3000 significant differential genes were found on each of the *Not3* knockdowns performed (Additional file 1: Tables S3–S6). Combining the different gene lists revealed 1028 misregulated genes, with 448 and 580 significantly up- and downregulated, respectively (Additional file 1: Table S7).

Gene set enrichment analysis (GSEA) identified DNA replication and ribosome biogenesis as significantly enriched signaling pathways (*p* = 4.81E−6 and *p* = 9.68E−4, respectively) (Fig. 5c). Notably, we found that 30 out of 34 genes from the DNA replication pathway were significantly upregulated (Fig. 5d). Metabolic, RNA transport and cell cycle signaling pathways were also highly enriched (Additional file 1: Table S8). Of importance, we only identified significantly enriched signaling pathways among the upregulated genes (Fig. 5e). These findings strongly support that the upregulated genes are specific effects of *Not3* knockdown, while downregulated genes may represent secondary effects.

Besides, we also detected upregulation of genes that could be linked to tumorigenesis. These included the pupation regulator insulin-like peptide 8 (*Ilp8*), the three unpaired (*upd*) genes (which encode the ligands for the JAK/STAT pathway), the cyclin B (*Cycb*) cell cycle regulator, and the discs proliferation abnormal (*dpa*) and the *PCNA* genes (Additional file 1: Table S7). Increased expression of those selected genes was further validated by using qPCR on the sensitized and the wild-type models. These results show that downregulation of *Not3* causes the upregulation of those genes specifically and independently of *Dl* ectopic expression (Fig. 5f).

In order to clarify whether Not3 regulates mRNA decay of those target genes or controls the transcription of those genes, we performed an i-CisTarget analysis on the upregulated genes to determine if Not3 DNA-binding sites were present in the promoters of the upregulated genes. We did not see any enrichment of the described Not3 motif [29] (NES = 0.72). Moreover, with PWMtools, we looked for occurrences of the Not3 motif in the promoter sites of these upregulated genes and we found that only a small portion of the genes, not more than expected by chance, has Not3 binding motifs. Next, we tested the stability of some target genes in vitro using Drosophila S2 cells (Additional file 5: Figure S4A–C). Our results show a clear knockdown of the *Not3* mRNA expression (almost 50% efficiency) at time point 0 h, which was associated with significant upregulation of *Cycb*, *fancl*, and *upd2* (Additional file 5: Figure S4B). No significant changes were detected on the expression level of *CG1239*, a gene we used as a control, since its expression was not significantly changed on our RNA-sequencing experiments after *Not3* downregulation (Additional file 5: Figure S4B). Analysis after transcription block provided a view on mRNA stability and changes induced by *Not3* downregulation. *Cycb*, *fancl*, and *upd2* mRNAs showed a moderate increase in stability after *Not3* downregulation when compared with the control condition (YFP expression). Again, no effects were observed on the mRNA stabilization of *CG1239* (Additional file 5: Figure S4C), indicating that the effects observed are specific. In conclusion, our results show that *Not3* downregulation causes an upregulation of *Cycb*, *fancl*, and *upd2* transcripts in S2 cells and that this can be linked to increased mRNA stability.

### Upregulation of CycB contributes to the Not3 tumor phenotype

We decided to investigate the function of seven selected target genes (*Cycb*, *os*, *upd2*, *upd3*, *Fancl*, *PCNA*, and *dpa*) on tumor development by knocking down their expression in the sensitized + *Not3-*tumor model (independent RNAi constructs/loss-of-function mutants were tested per gene). Before doing that, we excluded the possibility that downregulation of the selected genes could already cause defects on the wild-type retina. Downregulation of *PCNA* and *dpa* led to significant developmental defects on the fly’s retina, preventing their study in the eye tumor models.

**Fig. 6:**
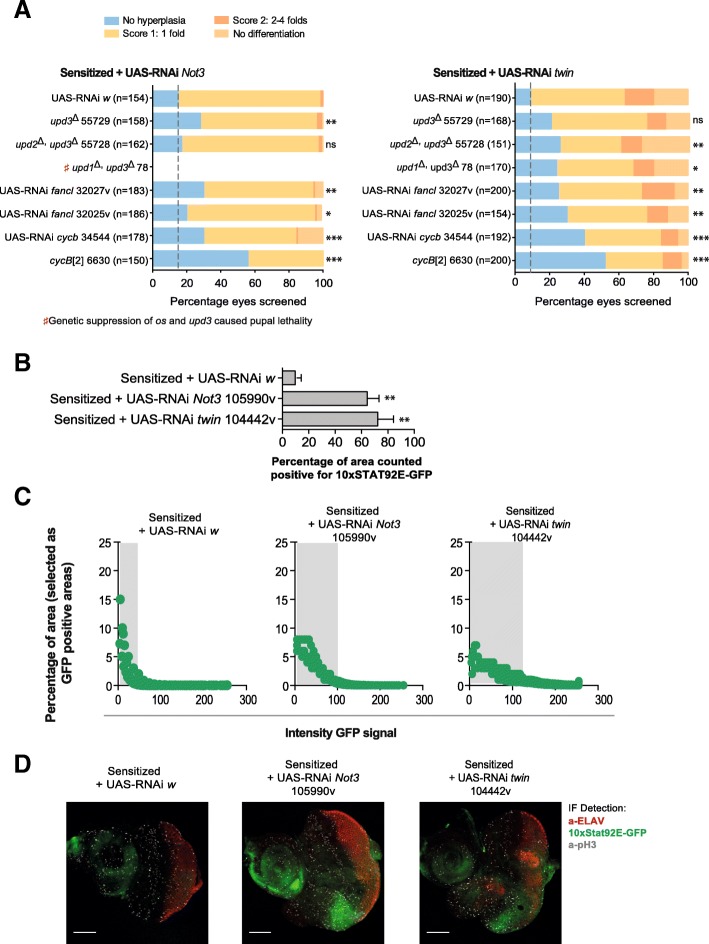
Upregulation of *cycB* contributes to the tumor phenotype in *Not3* and *twin* sensitized tumor models. **a** Sensitized + *Not3* defective tumor model and sensitized + *twin* defective tumor model. Bar graphs showing rescue scoring results for tumor model or tumor plus knockdown for the indicated gene. Two independent RNAis/mutant lines are shown. Three independent crosses were screened on each experimental condition. The mean number of eyes analyzed is shown on the graph (*Y*-axis). Genetic suppression of *os* and *upd3* on the sensitized + Not3 tumor animals caused pupal lethality precluding its analysis. Blue, no hyperplasia; yellow, score 1: 1 fold; light orange, score 2: 2–4 folds; dark orange, no differentiation; gray, no data available. ns, not significant; * *p* < 0.05; ** *p* < 0.01; *** *p* < 0.001. **b** Quantification of 10xSTAT92E-GFP expression domains on the posterior part of L3 wandering larval eye-antennal discs using the color inspector 3D ImageJ plugin. GFP expression domains were measured by analysis of confocal micrographs and expressed as a percentage of the posterior eye-disc area. Bar graphs show the percentage of areas positive for GFP signal (thus, areas with JAK-STAT signaling activation) from the different genotypes; ** *p* < 0.01. Six eye-antennal discs were analyzed per experimental condition. Experimental crosses were performed in triplicate. Graph shows results from one representative experiment. **c** Dot graphs show the green signal intensity of those areas previously selected as GFP positive areas. We observed that both the area of GFP expression domains and the green intensity of those areas are increased in sensitized + *Not3* (*n* = 6) or sensitized + *twin* tumor eye-antennal discs (*n* = 6). Experimental crosses were performed in triplicate; graph shows results from one representative experiment. **d** Representative confocal images of eye-antennal imaginal discs of the indicated genotypes. Green signal: 10xSTAT92E-GFP; red signal: ELAV protein; white signal: pH3 positive cells. Scale bars are 50 μM

For the other genes, we quantified effects on eye tumor rescue. Knockdown of *CycB* on the sensitized + *Not3*-tumor model yielded the strongest effects. The *cycB* [2] mutant rescued the tumor phenotype to over 50% compared to the control RNAi construct (~ 15% for *white* RNAi). Knockdown of *fancl* and JAK-STAT ligands had minor effects. For instance, knockdown of *upd2/upd3* showed rescue of tumor formation in 28% of eyes screened. A trend towards significance (*p* = 0.06) was observed on tumor rescue upon knockdown of *upd3* only (Fig. 6a). Genetic suppression of *os* and *upd3* on the sensitized + *Not3*-tumor animals caused pupal lethality, precluding its analysis. If tumor development associated with *Not3* disruption is linked to the CCR4-NOT complex functionality, downregulation of selected genes could also rescue tumor development in the sensitized + *twin*-tumors. Again, genetic suppression of *Cycb* yielded the strongest effects, with the *cycb* [2] mutant rescuing tumor phenotype to over 50% compared to the control RNAi construct (~ 10% for white RNAi) (Fig. 6a). Knockdown of *fancl* and genetic suppression of the JAK-STAT ligands on the sensitized + *twin* background showed a significant effect on tumor rescue (Fig. 6a).

Examination of expression of a 10xSTAT92E-GFP reporter for JAK-STAT pathway activation [30] by immunofluorescence upon *Not3* or *twin* downregulation on the sensitized model was then performed. We observed a significant increase of the 10xSTAT92E-GFP expression domains (Fig. 6b–d). However, since knockdown of JAK-STAT ligands had minor effects on tumor growth rescue, especially upon *Not3* downregulation, it might be that the pathway is active due to alternative mechanisms.

Taken together, our results show that upregulation of *CycB* contributes to the tumor phenotype in our sensitized *Not3* and *twin* tumor models.

### Loss of CNOT3 leads to stabilization of DNA replication and ribosome biogenesis signaling components in human T-ALL cell lines

**Fig. 7:**
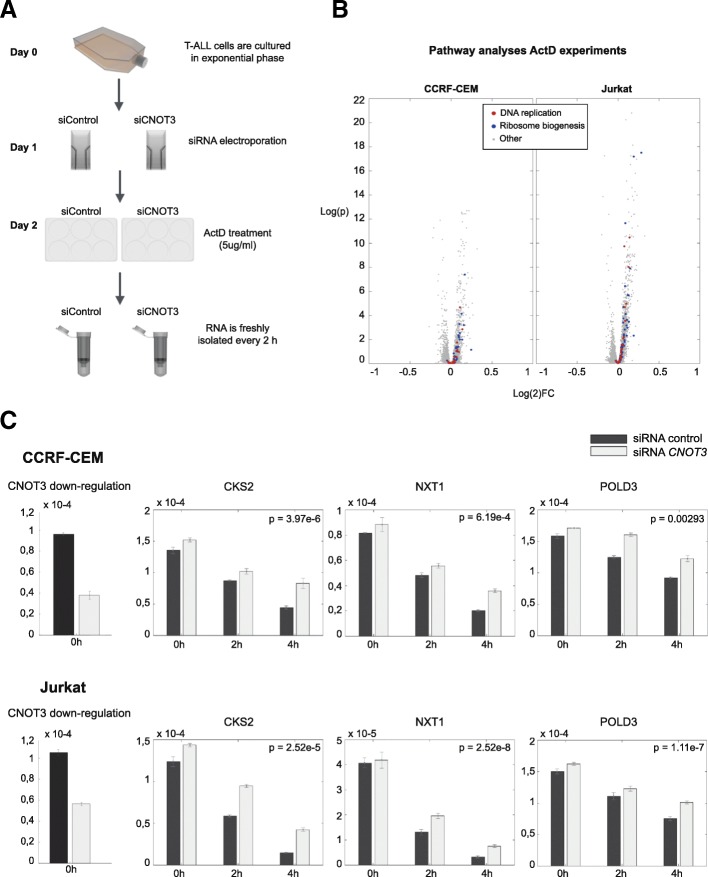
Loss of CNOT3 leads to stabilization of DNA replication and ribosome biogenesis signaling components in human T-ALL cell lines. **a** Workflow for the Actinomycin D experiments upon CNOT3 downregulation on T-ALL cell lines. Experiments were performed in three independent series. **b** Volcano plots from Jurkat and CCRF-CEM T-ALL cell lines. Those genes belonging to one of the most enriched signaling pathways are marked in color. **c** mRNA expression levels of CNOT3 in all conditions and mRNA expression levels of three exemplary genes that become more stable upon CNOT3 knockdown, i.e., they show an increasing difference between the knockdown and the wild-type. Experiments were performed in three independent series

Thereafter, we tested if the results obtained in Drosophila could be translated to human T-ALL, since CNOT3 mutations were described in this tumor type. We knocked down *CNOT3* in human T-ALL cell lines JURKAT and CCRF-CEM by using siRNAs (two *CNOT3* specific and a negative control siRNA). We determined effects on steady-state mRNA expression levels and also assessed the effect on RNA stability by blocking transcription and measuring the half-life of mRNA’s in a global way by RNA-seq. We stopped the transcription of newly synthesized mRNA by treating cells with Actinomycin D 24 h after electroporation. RNA was isolated at 0 h (reference time point), 2 h, and 4 h time points after Actinomycin D treatment and submitted to RNA-sequencing (Fig. 7a).

As expected, we observed a clear knockdown of the CNOT3 mRNA expression (almost 50% efficiency) at time point 0 h (Fig. 7b), which was associated with the upregulation of 975 and 1611 transcripts in JURKAT and CCRF-CEM cells, respectively (Additional file 1: Tables S9 and S10). Pathway analysis with DAVID revealed that, similar to our findings in Drosophila, the DNA replication pathway and the ribosome biogenesis pathways were significantly enriched (Fig. 7c, Additional file 1: Tables S11–S13) in both cell lines. RNA-seq analysis at 2 h and 4 h after transcription block provided a genome-wide view on mRNA stability and changes induced by CNOT3 downregulation. Among the significantly upregulated and more stable genes were genes involved in DNA replication pathway and the ribosome biogenesis including *NXT1*, *POLD3*, and *CKS2* (Fig. 7c, Additional file 1: Tables S12 and S13).

## Discussion

Mutations on CNOT3, and more rarely in other members of the CCR4-NOT complex, have recently been identified in T-ALL and chronic lymphoblastic leukemia [13, 31]. In solid tumors, recent reports show that CNOT3 and other CCR4-NOT subunits could be involved in tumor formation/progression. Downregulation of *Cnot2* expression results in a significant increase of breast cancer pulmonary metastasis in vivo [32]. Instead, *CNOT3* confers an aggressive behavior in colorectal cancer cells through a self-renewal transcriptional program [33]. Mutations on *CNOT3* have been found in T-ALL patients experiencing treatment failure after first relapse, although this observation needs to be confirmed in a larger cohort of patients [34]. Thus, it seems that depending on the cellular context, those proteins can have either tumor suppressor or oncogenic properties. However, it still remains unclear how mutations on CNOT3 or other CCR4-NOT subunits can contribute to tumor development.

In this report, we show that *Not3* behaves as a classical tumor suppressor, with reduction of *Not3* expression leading to increased tumor formation and overexpression of *Not3* leading to suppressed tumor formation. Interestingly, and in agreement with sequencing data in human cancers, we also observed increased tumor formation upon knockdown of other members of the CCR4-NOT complex, including the deadenylases. These data make clear that the entire CCR4-NOT complex functions as a tumor suppressor complex and that this is likely, at least in part, through its function in mRNA metabolism.

In human cancer, only one allele of CNOT3 is mutated, suggesting that this is sufficient to contribute to tumor development and that complete loss of CNOT3 is not viable for the cells. In fact, our results show that little reduction on *Not3* expression levels in a sensitized/pre-neoplastic background is sufficient to initiate and dramatically increase tumor formation. Conversely, a little reduction on *Not3* expression is not enough to cause defects on wild-type tissues. This is consistent with previous data showing that the presence of only one *Cnot3* allele is enough to sustain normal cell development [6].

Recent structure-function analyses have revealed that CNOT2 and CNOT3 bind to the CCR4-NOT complex through the Not-box domain [27, 28]. We demonstrate that overexpression of a *Not3* mutant lacking the Not-box domain does not rescue neither the *Not3*-downregulated nor the eyeful tumor phenotype. These data confirm that the defects observed in our models are related to the function of *Not3* within the CCR4-NOT complex. In agreement, it has been shown that expression of a CNOT3 mutant lacking the Not-box domain in CNOT3-depleted mouse embryonic fibroblasts was not able to rescue proper formation of the CCR4-NOT complex and the decreased cell viability [35].

To further determine the exact consequences of downregulation of *Not3*, we performed RNA-sequencing on Drosophila eye discs. CNOT3 was described to be involved in transcriptional regulation [26, 32, 35]. However, we did not find evidence that Not3 is directly involved in the transcription of the genes that were upregulated upon Not3 knockdown, as the Not3 DNA-binding motif was not enriched in the promoters of that gene set. Our findings revealed an important role for Cyclin B (*CycB*), a gene known to be upregulated in various cancers [36, 37]. We observed that *CycB* was upregulated upon *Not3* downregulation and stabilized upon *Not3* downregulation in S2 cells in vitro. Moreover, downregulation of *CycB* counteracted tumor formation, illustrating an important role for *CycB* in these tumors.

Our RNA-sequencing analyses also revealed that genes involved in DNA replication and ribosome biogenesis are significantly increased upon *Not3* downregulation. Moreover, we confirmed on human cell lines that the increased mRNA expression level of those genes is due to mRNA stabilization, and not to a significant increased on gene transcription rates. Increased expression of genes involved in DNA replication has been found in cancer cells [38] and might result in increased replication initiation activity at a global level. There is growing evidence that an upregulated ribosome biogenesis might provide an increased risk of cancer onset [39]. Aberrant ribosome synthesis contributes to increased cellular proliferation [40, 41]. Differential expression of several ribosomal protein genes has been observed in cancer [39, 42]. Thus, *Not3/CNOT3* loss could contribute to cancer development through those two signaling pathways.

## Conclusions

- We establish for the first time that *Not3* and other members of the CCR4-NOT complex act as tumor suppressor genes.
- Our results indicate that the entire CCR4-NOT complex serves as a tumor suppressor, in part by suppressing transcripts implicated in DNA replication and RNA biogenesis.

## Additional files

Additional file 1: **Table S1.** Fly stocks used in the present study. Table S2 Primers used for cloning and real-time PCR. Table S3 Differential gene analysis. Comparison using the sensitized + UAS-RNAi Not3 105990v as target. Table S4 Differential gene analysis. Comparison using the sensitized + UAS-RNAi Not3 37547v as target. Table S5 Differential gene analysis. Comparison using the sensitized + UAS-RNAi Not3 37545v as target. Table S6 Differential gene analysis. Comparison using the sensitized + Not3 P-element as target. Table S7 List of core differential genes in all 4 comparisons. Table S8 Pathway analyses derived from the gene eye tumor signature, results from a FlyMine analysis. Table S9. Differential genes in Actinomycin D experiments on JURKAT cells. Table S10 Differential genes in Actinomycin D experiments on CCRF-CEM cells. Table S11 Description of the KEGG gene sets. Table S12 KEGG analyses on JURKAT cells. Table S13 KEGG analyses on CCRF-CEM cells. (XLSX 14974 kb)

Additional file 2: **Figure S1.** Reduced expression levels of twin lead to a change on the number of positive pH3 on eye discs from ey-Gal4 wild type and sensitized fly models. Quantification of the number of pH3 positive cells on the posterior portion of eye-antennal imaginal discs (*n* = 10) from sensitized + *twin* larvae. Representative confocal images of eye-antennal imaginal discs of the indicated genotypes. Green staining: BrdU, pH3 or cleaved-DCP1a positive cells; red staining: ELAV protein. Results are compared with data of the other genotypes shown in Figs. 2c and 4a. Scale bars are 70 μM. (PDF 4225 kb)

Additional file 3 Figure S2. Expression of the different CCR4-NOT subunits after downregulation on ey-Gal4 wild-type and sensitized fly models. Downregulation of Not1, Not2, Pop2, and twin on wild-type eye-antennal discs and B) Downregulation of Not1, Not2, Pop2, and twin on sensitized eye-antennal discs. Bars represent expression mRNA levels, normalized using Rps13 as house-keeping gene and ey-Gal4 (wild-type) + UAS-RNAi white as reference sample. Each bar represents pool of 40 eye-antennal discs isolated from 40 L3 wandering larvae from two independent crosses. (PDF 390 kb)

Additional file 4: **Figure S3.** Similar expression levels are achieved and translated proteins are equally stable when we ectopically express the different Not3 constructs. A) The yellow graph bar represents the percentage of transfection achieved on S2 cells. Western blot analyses show protein levels of the different Not3 proteins 24 h after gene expression induction, with or without cycloheximide treatment. No differences on protein expression/stability among the UAS-Not constructs were observed. Asterisks indicate unspecific protein bands. B) qPCR analyses showing the Not3 expression levels on each genotype. Each bar represents the expression value of a pool of 10 individuals. (PDF 422 kb)

Additional file 5: **Figure S4.** Stabilization of mRNA expression levels of Cycb, fancl, and upd2 upon Not3 downregulation and transcription inhibition on Drosophila S2 cells in vitro. **A**) Percentage of YFP expressing cells upon addition of CuSO4 on the cell media. **B**) Expression of the different genes at time-point 0. Our results show a clear knockdown of the Not3 mRNA expression (almost 50% efficiency) at time point 0 h, which was associated with upregulation of Cycb, fancl, and upd2. **C**) Cycb, fancl, and upd2 mRNAs showed a moderate increase in stability after Not3 downregulation when compared with the control condition (YFP expression). In all figure panels, results are shown as mean ± S.D. Three independent experiments were performed. (PDF 418 kb)

## Abbreviations

CC: Coiled coil
Cycb: Cyclin B
Dl: Notch ligand Delta
dpa: Discs proliferation abnormal
GSEA: Gene set enrichment analysis
NB: Not-box
pH3: Phospho-Histone3
T-ALL: T-cell acute lymphoblastic leukemia
upd: Unpaired
