# Supplementary figures and images for: The CCR4-NOT complex is a tumor suppressor in Drosophila melanogaster eye cancer models
Source: J Hematol Oncol. 2018 Aug 25;11:108. doi: 10.1186/s13045-018-0650-0 (PMC6109294; doi:10.1186/s13045-018-0650-0)

Figure S1

A

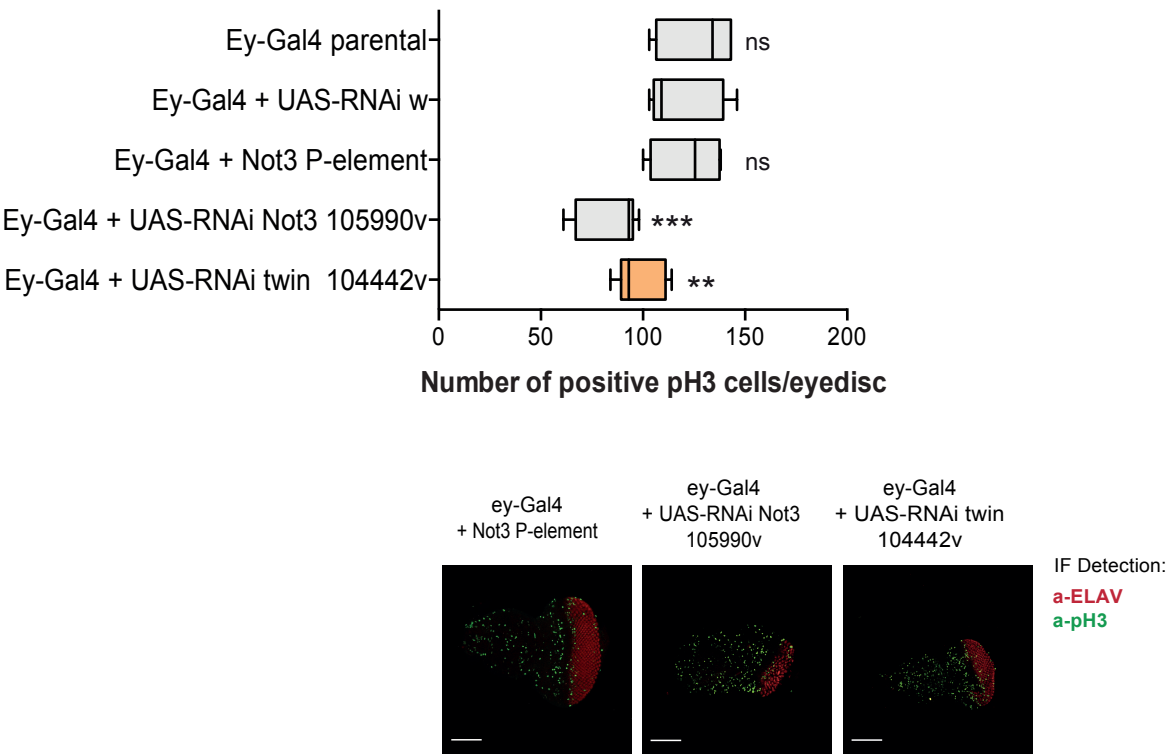

B

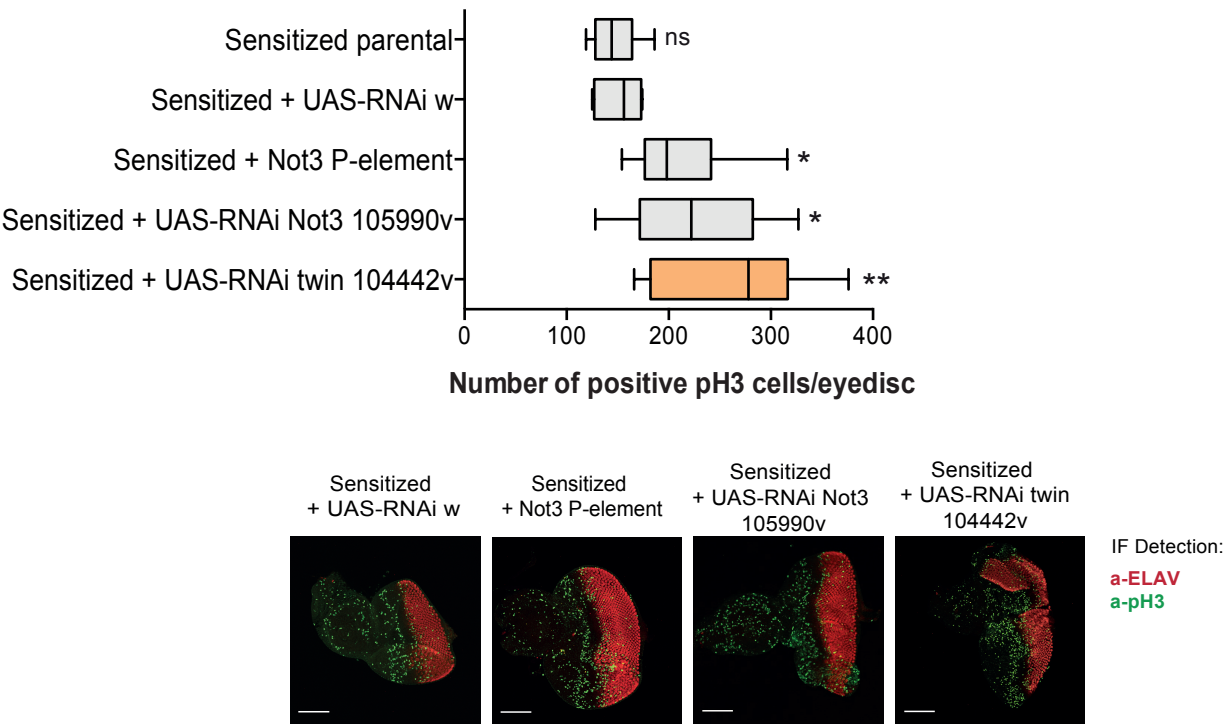

Supplement: Supplementary file 2 — Figure S1. Reduced expression levels of twin lead to a change on the number of positive pH3 on eye discs from ey-Gal4 wild type and sensitized fly models. Quantification of the number of pH3 positive cells on the posterior portion of eye-antennal imaginal discs (n = 10) from sensitized + twin larvae. Representative confocal images of eye-antennal imaginal discs of the indicated genotypes. Green staining: BrdU, pH3 or cleaved-DCP1a positive cells; red staining: ELAV protein. Results are compared with data of the other genotypes shown in Figs. 2c and 4a. Scale bars are 70 μM. (PDF 4225 kb) [file 13045_2018_650_MOESM2_ESM.pdf]

Figure S3

A

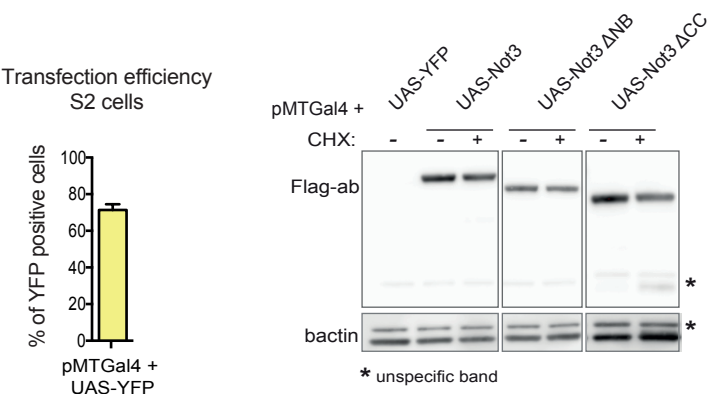

B

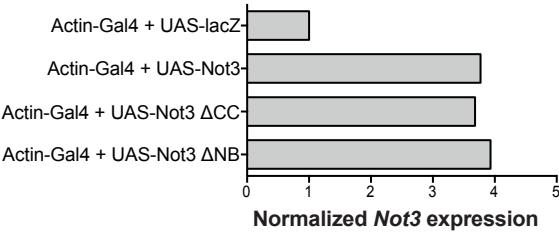

Supplement: Supplementary file 4 — Figure S3. Similar expression levels are achieved and translated proteins are equally stable when we ectopically express the different Not3 constructs. A) The yellow graph bar represents the percentage of transfection achieved on S2 cells. Western blot analyses show protein levels of the different Not3 proteins 24 h after gene expression induction, with or without cycloheximide treatment. No differences on protein expression/stability among the UAS-Not constructs were observed. Asterisks indicate unspecific protein bands. B) qPCR analyses showing the Not3 expression levels on each genotype. Each bar represents the expression value of a pool of 10 individuals. (PDF 422 kb) [file 13045_2018_650_MOESM4_ESM.pdf]

Figure S4

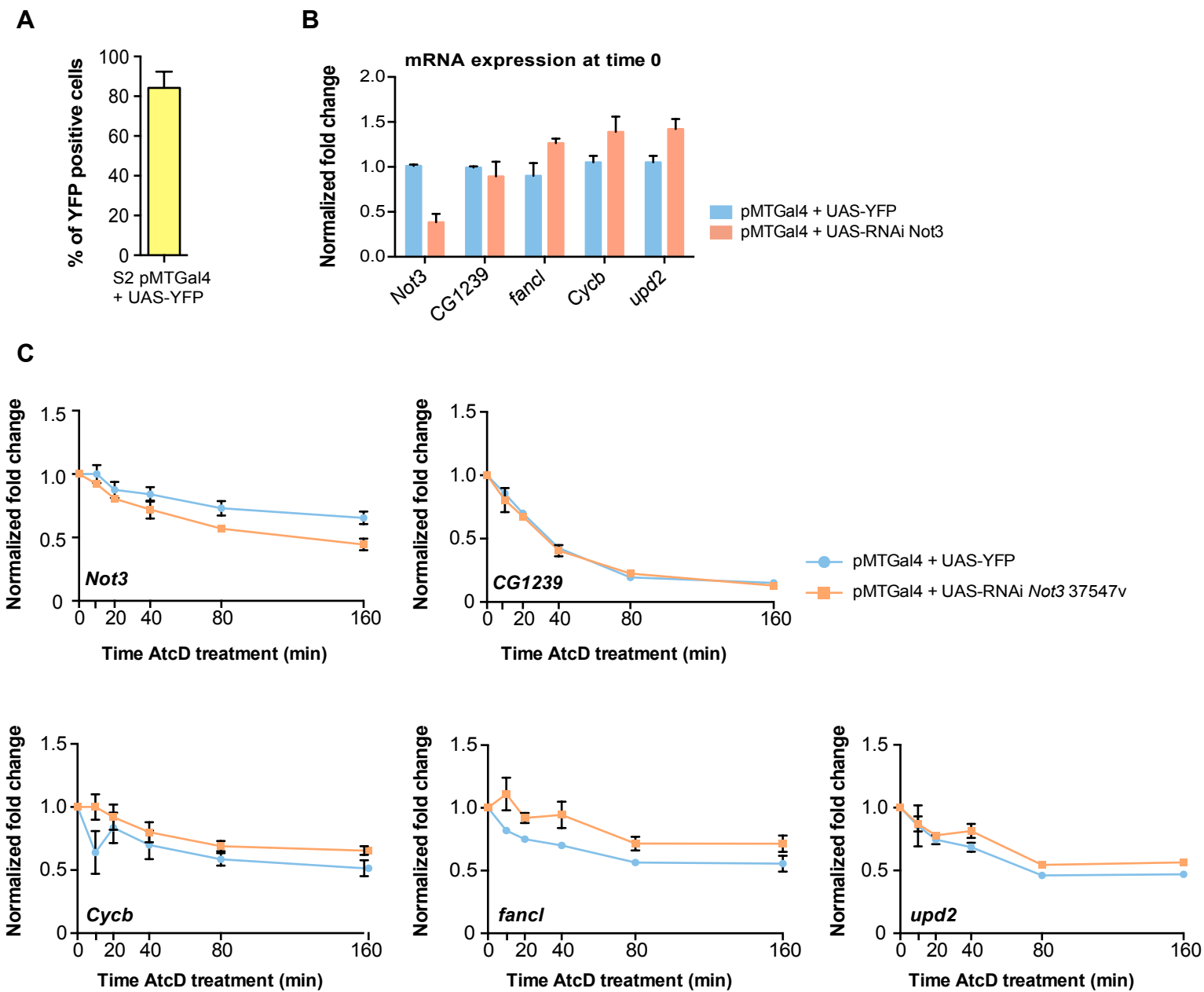

Supplement: Supplementary file 5 — Figure S4. Stabilization of mRNA expression levels of Cycb, fancl, and upd2 upon Not3 downregulation and transcription inhibition on Drosophila S2 cells in vitro. A) Percentage of YFP expressing cells upon addition of CuSO4 on the cell media. B) Expression of the different genes at time-point 0. Our results show a clear knockdown of the Not3 mRNA expression (almost 50% efficiency) at time point 0 h, which was associated with upregulation of Cycb, fancl, and upd2. C) Cycb, fancl, and upd2 mRNAs showed a moderate increase in stability after Not3 downregulation when compared with the control condition (YFP expression). In all figure panels, results are shown as mean ± S.D. Three independent experiments were performed. (PDF 418 kb) [file 13045_2018_650_MOESM5_ESM.pdf]
