# Supplementary material for: The CCR4-NOT complex is a tumor suppressor in Drosophila melanogaster eye cancer models
Source: J Hematol Oncol. 2018 Aug 25;11:108. doi: 10.1186/s13045-018-0650-0 (PMC6109294; doi:10.1186/s13045-018-0650-0)
Supplement: Supplementary file 3 — Figure S2. Expression of the different CCR4-NOT subunits after downregulation on ey-Gal4 wild-type and sensitized fly models. Downregulation of Not1, Not2, Pop2, and twin on wild-type eye-antennal discs and B) Downregulation of Not1, Not2, Pop2, and twin on sensitized eye-antennal discs. Bars represent expression mRNA levels, normalized using Rps13 as house-keeping gene and ey-Gal4 (wild-type) + UAS-RNAi white as reference sample. Each bar represents pool of 40 eye-antennal discs isolated from 40 L3 wandering larvae from two independent crosses. (PDF 390 kb) [file 13045_2018_650_MOESM3_ESM.pdf]

Figure S2

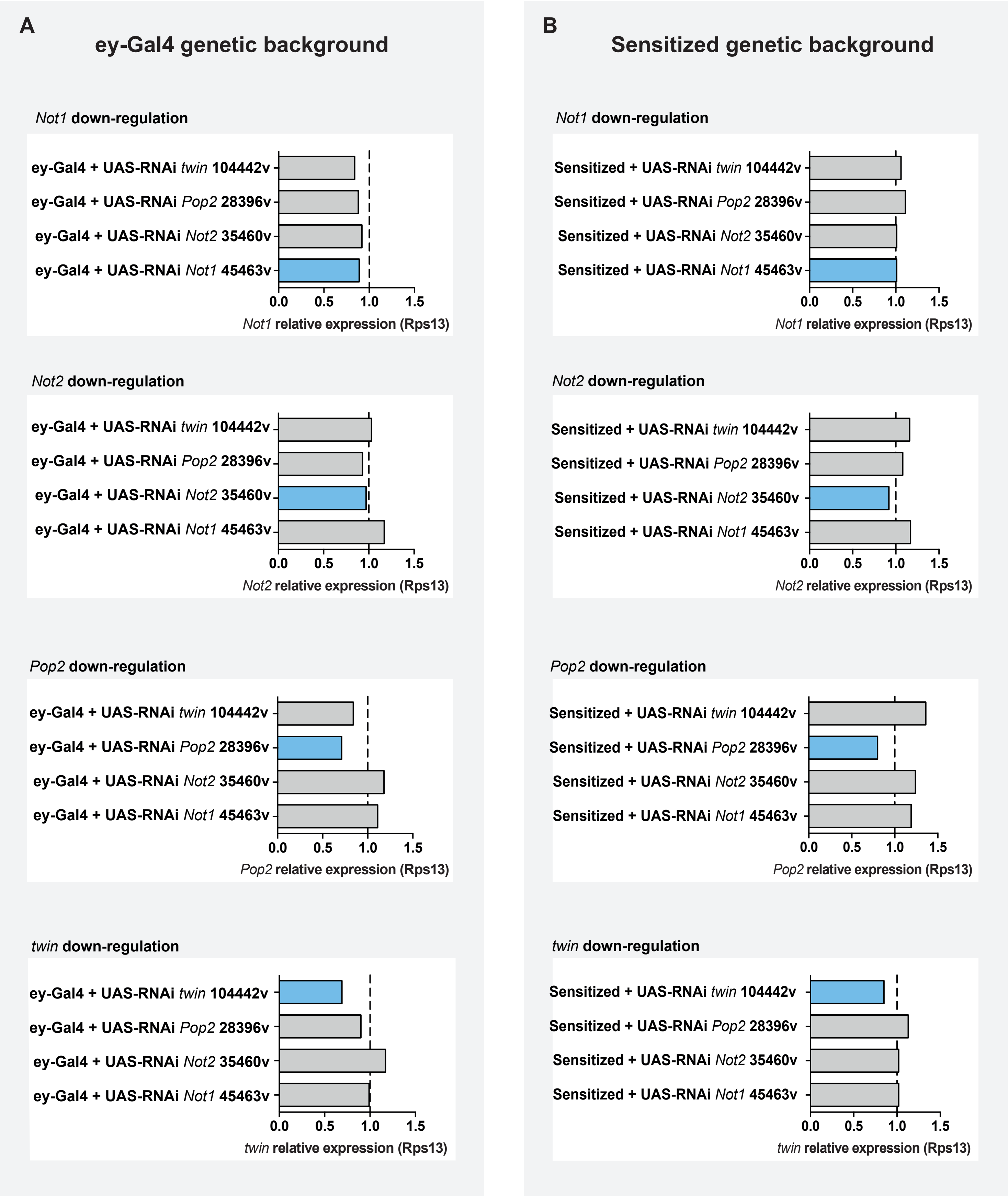
A
ey-Gal4 genetic background
Not1 down-regulation
ey-Gal4 + UAS-RNAi twin 104442v
ey-Gal4 + UAS-RNAi Pop2 28396v
ey-Gal4 + UAS-RNAi Not2 35460v
ey-Gal4 + UAS-RNAi Not1 45463v
0.0 0.5 1.0 1.5
Not1 relative expression (Rps13)
Not2 down-regulation
ey-Gal4 + UAS-RNAi twin 104442v
ey-Gal4 + UAS-RNAi Pop2 28396v
ey-Gal4 + UAS-RNAi Not2 35460v
ey-Gal4 + UAS-RNAi Not1 45463v
0.0 0.5 1.0 1.5
Not2 relative expression (Rps13)
Pop2 down-regulation
ey-Gal4 + UAS-RNAi twin 104442v
ey-Gal4 + UAS-RNAi Pop2 28396v
ey-Gal4 + UAS-RNAi Not2 35460v
ey-Gal4 + UAS-RNAi Not1 45463v
0.0 0.5 1.0 1.5
Pop2 relative expression (Rps13)
twin down-regulation
ey-Gal4 + UAS-RNAi twin 104442v
ey-Gal4 + UAS-RNAi Pop2 28396v
ey-Gal4 + UAS-RNAi Not2 35460v
ey-Gal4 + UAS-RNAi Not1 45463v
0.0 0.5 1.0 1.5
twin relative expression (Rps13)
B
Sensitized genetic background
Not1 down-regulation
Sensitized + UAS-RNAi twin 104442v
Sensitized + UAS-RNAi Pop2 28396v
Sensitized + UAS-RNAi Not2 35460v
Sensitized + UAS-RNAi Not1 45463v
0.0 0.5 1.0 1.5
Not1 relative expression (Rps13)
Not2 down-regulation
Sensitized + UAS-RNAi twin 104442v
Sensitized + UAS-RNAi Pop2 28396v
Sensitized + UAS-RNAi Not2 35460v
Sensitized + UAS-RNAi Not1 45463v
0.0 0.5 1.0 1.5
Not2 relative expression (Rps13)
Pop2 down-regulation
Sensitized + UAS-RNAi twin 104442v
Sensitized + UAS-RNAi Pop2 28396v
Sensitized + UAS-RNAi Not2 35460v
Sensitized + UAS-RNAi Not1 45463v
0.0 0.5 1.0 1.5
Pop2 relative expression (Rps13)
twin down-regulation
Sensitized + UAS-RNAi twin 104442v
Sensitized + UAS-RNAi Pop2 28396v
Sensitized + UAS-RNAi Not2 35460v
Sensitized + UAS-RNAi Not1 45463v
0.0 0.5 1.0 1.5
twin relative expression (Rps13)
